# The Type III Secretion Effector NleE Inhibits NF-κB Activation

**DOI:** 10.1371/journal.ppat.1000743

**Published:** 2010-01-29

**Authors:** Chen Nadler, Kobi Baruch, Simi Kobi, Erez Mills, Gili Haviv, Marganit Farago, Irit Alkalay, Sina Bartfeld, Thomas F. Meyer, Yinon Ben-Neriah, Ilan Rosenshine

**Affiliations:** 1 Department of Microbiology and Molecular Genetics, IMRIC, Faculty of Medicine, The Hebrew University, Jerusalem, Israel; 2 Department of Immunology and Cancer Research, IMRIC, Faculty of Medicine, The Hebrew University, Jerusalem, Israel; 3 Department Molecular Biology, MPI for Infection Biology, Berlin, Germany; Stanford University, United States of America

## Abstract

The complex host-pathogen interplay involves the recognition of the pathogen by the host's innate immune system and countermeasures taken by the pathogen. Detection of invading bacteria by the host leads to rapid activation of the transcription factor NF-κB, followed by inflammation and eradication of the intruders. In response, some pathogens, including enteropathogenic *Escherichia coli* (EPEC), acquired means of blocking NF-κB activation. We show that inhibition of NF-κB activation by EPEC involves the injection of NleE into the host cell. Importantly, we show that NleE inhibits NF-κB activation by preventing activation of IKKβ and consequently the degradation of the NF-κB inhibitor, IκB. This NleE activity is enhanced by, but is not dependent on, a second injected effector, NleB. In conclusion, this study describes two effectors, NleB and NleE, with no similarity to other known proteins, used by pathogens to manipulate NF-κB signaling pathways.

## Author Summary

The innate immune system senses intruding pathogens and in response, mounts an inflammatory reaction. Essential for this response is the activation of the transcription factor NF-κB, which mediates reprogramming of gene expression in the host. The bacteria *Escherichia coli* is usually a non-pathogenic resident of our intestinal track. Some *E. coli* strains, however, cause disease or food poisoning; one of these pathogenic strains is enteropathogenic *E. coli* (EPEC). This pathogen employs a syringe-like organelle, termed type three secretion system (TTSS), to inject into the intestinal host cell a battery of toxic proteins termed effectors. We found that two of the effectors that EPEC injects into the host cell upon infection block the activation of NF-κB and thus interfere with the host immune response. These findings elucidate the intricate cross-talk between the host immune system and the pathogen.

## Introduction

Enteropathogenic *Escherichia coli* (EPEC) belong to a group of pathogens defined by their ability to form “attaching and effacing” (AE) histopathology on intestinal epithelia. These pathogens employ their type III protein secretion system (TTSS) to inject (translocate) toxic proteins (effectors) into the host cell. The injected effectors subvert normal host cell functions to benefit the bacteria (summarized in [1]). To date, 21 effectors or putative effector genes have been described for EPEC. Six of them are encoded in the LEE region that also encodes the TTSS structural genes, whereas the other 15 effector genes are distributed within three prophages and three insertion elements (IE) [2].

Upon infection, bacterial PAMPs (pathogen-associated molecular patterns) including LPS, flagellin, lipoproteins, and CpG DNA stimulate host cell Toll-like receptors (TLRs) in the host cells, leading to a formidable immune response via the activation of the transcription factor NF-κB [3],[4]. NF-κB comprises a family of closely related transcription factors that play a key role in the expression of genes involved in inflammation, immune, and stress responses. NF-κB is a collective term used for homo- and heterodimeric complexes formed by the Rel/NF-κB proteins. In mammals, five of such proteins are known: RelA (p65), RelB, c-Rel, p50 (NF-κB1), and p52 (NF-κB2). Under nonstimulating conditions, NF-κB is retained in the cytoplasm through its association with inhibitory proteins (IκBs). A variety of signaling pathways activate IκB kinases (IKK) to phosphorylate IκB, leading to its ubiquitination and degradation by the proteasome. This allows translocation of NF-κB to the nucleus, activation of NF-κB-regulated genes, and establishment of an inflammatory response [5],[6].

Previous reports have suggested that during infection, EPEC manipulate NF-κB-mediated inflammation. Initially, it was shown that EPEC activate NF-κB by a TTSS-dependent mechanism [7],[8], but later, it was reported that the TTSS is not required and that EPEC activate NF-κB via a TTSS-independent mechanism, presumably by activation of TLRs [9],[10],[11],[12]. Moreover, some reports showed that EPEC actually repress NF-κB activation by a TTSS-dependent mechanism [13],[14]. Taken together, these reports suggest that EPEC first elicit NF-κB activation by a TTSS-independent mechanism and subsequently, it utilize the TTSS mechanism to mediate TTSS-dependent NF-κB-repression. However, the major gap in the above hypothesis is that the putative effector that presumably represses NF-κB activation has never been identified. In this report we confirm that EPEC block NF-κB activation via a TTSS-dependent mechanism and show that the NleE effector is necessary and sufficient to block NF-κB activation via inhibition of IκB phosphorylation and thus induces its stabilization. In addition, we show that a second effector, NleB, is required for better repression of NF-κB activation, suggesting that the function of NleB is related to that of NleE.

## Results

### EPEC inhibit IκB degradation and NF-κB activation by a TTSS-dependent mechanism

**Figure 1:**
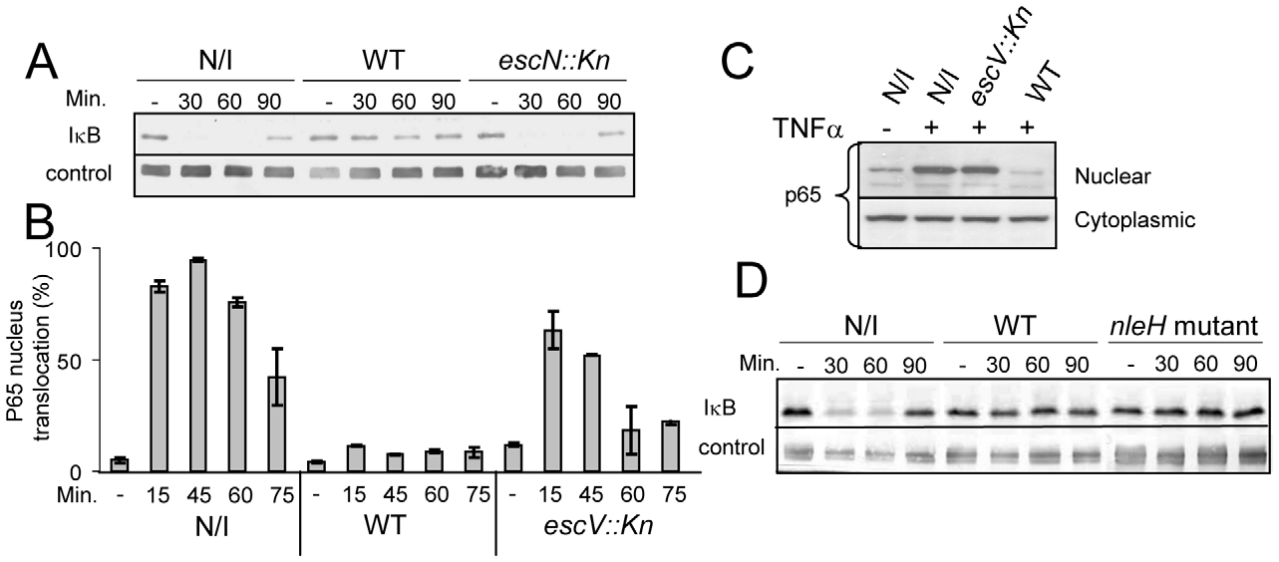
EPEC inhibit TNFα-induced IκB degradation and NF-κB translocation to the nucleus in a TTSS-dependent manner. (A) HeLa cells were infected with wild-type EPEC (WT), a TTSS mutant (*escN::kan*) or remained uninfected (N/I). After 3h, cells were washed and treated with 10 ng/ml TNFα. At the indicated time post TNFα treatment, cells were extracted and subjected to western blot analysis with anti-IκB antibodies and anti-tubulin (loading control). (B) AGS SIB02 cells expressing p65-GFP were infected with wild-type EPEC or with a EPEC TTSS mutant (*escV::kan*) or remained uninfected. After 3 h, the cells were washed and treated with TNFα. At the indicated time post TNFα treatment, cells were fixed, nuclei stained, and the levels of p65-GFP translocation in ∼200 cells were analyzed by automated microscopy and image analysis software. Standard errors are indicated by bars. (C) HeLa cells were infected with different strains and treated with TNFα as described for (B). At 30 min post TNFα induction, cells were harvested and fractionated into nuclear and cytoplasmic fractions. The presence of the p65 in the different fractions was analyzed by Western blot using anti-p65 antibody. (D) IκB degradation assay was conducted (as described for A) using uninfected cells, cells infected with EPEC wild-type strain and with an EPEC mutant deleted of its two *nleH* alleles (*nleH* mutant).

The ability of EPEC to either inhibit or induce NF-κB activation is controversial. Therefore, we re-examined this point using HeLa cells as host cells and IκB stability as a read-out for NF-κB activation. Importantly, TNFα treatment strongly stimulates IκB degradation in these cells, but under the used experimental conditions they exhibited minimal IκB degradation upon exposure to the PAMPs of the infecting EPEC. This allowed the uncoupling of infection and NF-κB activation. HeLa cells were infected with EPEC culture for 3 h, during which the bacteria injected the TTSS effectors into host cells. The infected cells were then treated with 10 ng/ml TNFα to activate NF-κB and at different time points post TNFα-induction, cellular lysates were subjected to western analysis. The results show that TNFα treatment induced rapid degradation of IκB in uninfected cells or cells infected with EPEC TTSS-deficient mutant (*escN::kan*). In contrast, IκB in cells infected with wild-type EPEC remained stable (Fig. 1A).

We next tested whether the stabilization of IκB by EPEC was associated with inhibition of NF-κB translocation to the nucleus. To monitor NF-κB activation, we used a reporter cell line (AGS SIB02) stably expressing the NF-κB subunit p65 fused to GFP. Cells were infected with wild-type EPEC or, as a negative control, with EPEC TTSS mutant (*escV::kan*). After 3 h of infection, cells were washed, induced with TNFα, and at 15, 45, 60, and 75 min post TNFα-induction, the cells were fixed, stained with Hoechst 33342, and analyzed by automated microscopy. Importantly, whereas wild-type EPEC repressed p65-GFP translocation to the nucleus, the TTSS *escV* mutant was strongly attenuated in this activity (Fig. 1B). To validate the above microscopic analysis we carried out identical experiment, but instead of using microscopy to analyze the cells we fractionated the cells into cytoplasmic and nucleus fractions and determined the amount of p65 in the different fractions by immunoblot using anti-p65 antibody. The results were in agreement with the microscopic analysis: wild type EPEC, but not the *escV* mutant, blocked translocation of p65 to the nucleus (Fig. 1C). Thus supporting the notion that EPEC inhibit NF-κB activation by a TTSS-dependent mechanism [13],[14], and suggesting that EPEC deliver into infected cells one or more effectors that inhibits NF-κB activation.

NleH has been proposed as such an effector since it is similar to OspG, a *Shigella* effector that inhibits NF-κB activation [15]. However, we found that an EPEC strain, in which both *nleH* alleles were deleted, still inhibited IκB degradation, similarly to wild-type EPEC (Fig. 1D), suggesting that NleH is not required for blocking IκB degradation under the experimental conditions used by us.

### NleE and NleB are required for IκB stabilization

**Figure 2:**
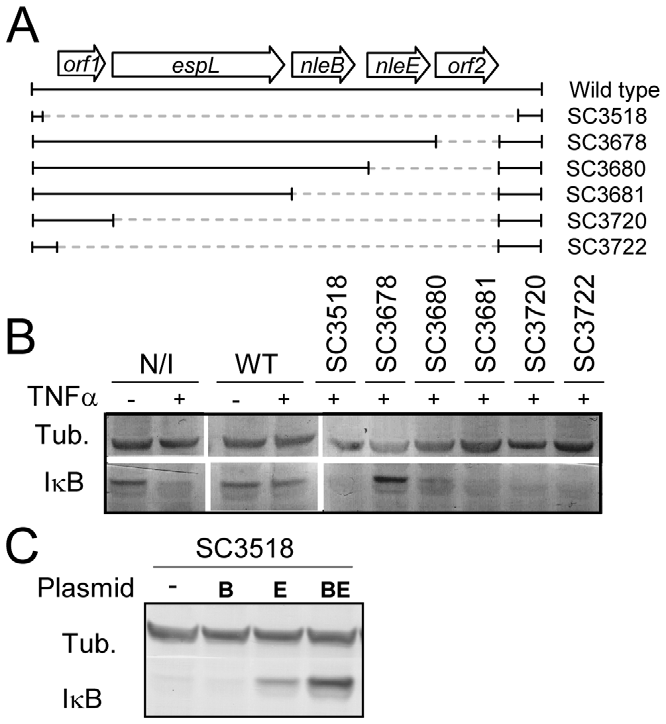
NleE is required for EPEC inhibition of TNFα-induced IκB degradation. (A) Schematic of the region within IE6 containing the *nleBE* genes and the chromosomal deletions used for the analysis. Black lines represent the chromosomal DNA and gray dashed lines represent the deleted regions. The corresponding names of the deleted strains are indicated on the right side. (B) HeLa cells were either uninfected (N/I) or infected with wild-type EPEC (WT) or with the different mutants shown in (A), as indicated. After 3h, cells were washed, treated with TNFα for 40 min and extracted. The extracts were analyzed by western blot with anti-IκB and anti-tubulin (loading control) antibodies. (C) HeLa cells were infected with a strain (SC3518) carrying a chromosomal deletion of the *nleBE* region, which was complemented or not complemented with plasmids expressing *nleB*, *nleE*, or *nleBE* (indicated as B, E, and BE, respectively). After 3h, cells were washed, treated with TNFα for 30 min. and extracted. The extracts were analyzed by western blot using anti-IκB and anti-tubulin antibodies.

To identify putative effector(s) that block IκB degradation, we bioinformaticly compared the genome of EPEC to that of non-pathogenic *E. coli* K12 and identified large EPEC-specific regions that contain, or possibly contain, effector genes. Based on this comparison, we constructed a set of 15 EPEC strains, each deleted of one EPEC-specific large chromosomal region (Table 1). Altogether, 770 EPEC-specific ORFs were deleted. We then tested the capacity of each of the deleted strains to inhibit IκB degradation upon TNFα treatment. One of the strains, deleted of the IE6 region [2], could not inhibit IκB degradation (data not shown). Further systematic deletion analysis defined two effector-encoding genes, *nleB* and *nleE*, required for stabilizing IκB (Fig. 2A, 2B and data not shown). Deletion of *nleE* strongly reduced the bacteria's capacity to stabilize IκB, but a complete deficiency in IκB stabilization was observed only in the strain deleted of both *nleB* and *nleE* (Fig. 2B). To corroborate the notion that NleE is required for IκB stabilization, we complemented a strain deleted of the *nleBE* region with plasmids containing *nleB*, *nleE*, or *nleBE*. We found that expression of NleE, but not of NleB, partially restored EPEC's capacity to stabilize IκB (Fig. 2C). Importantly, full IκB protection was achieved in strains expressing both NleB and NleE (Fig. 2C). A mutant expressing only NleB showed only low level of IκB protection (Fig. 2C). Taken together, these results suggest that NleB and NleE, located at the IE6 region, are necessary for stabilizing IκB and that this activity is contributed mainly by NleE (Fig. 2B). We therefore focused our attention on NleE.

**Table 1:** List of the deletions of large EPEC chromosomal islands.

Strain Name	Island Name^a^	Size in Kb	Location	Number of Putative ORFs	Notes^b^
EM3321	6M (PP2)	53.7	720891..774629	84	Contains *nleH*_pp2_, *espJ*, and a *cif* pseudogene
EM3323	7M	34.3	855103..889419	61	
EM3325	8M (PP4)	45	1046278..1091304	68	Contains *nleG/*, *nleB*_pp4_, *nleC*, an *nleH* fragment, and *nleD*
EM3327	9M (IE2)	61.2	1121572..1182762	60	Contains *lifA*/*efa1*_IE2_, *nleE*_IE2_, *nleB*_IE2_, and an *espL* pseudogene
EM3329	10M	40.6	1270890..1311501	50	
EM3331	13M (PP6)	55.6	1430190..1485767	61	Contains *nleH*_pp6_, *nleA/espI*, *nleF*, and an *espO* fragment
EM3333	17M	36.4	1869528..1905895	55	
EM3335	18Ma	26.4	2149581..2175998	30	
EM3337	18Mb	18	2183694..2201688	30	
EM3339	21M	47.8	2590597..2638364	71	
EM3341	22M	36.6	2718268..2754895	55	
EM3343	24M	58.9	2814904..2873848	72	
EM3345	26M (IE5)	15.2	3001568..3016789	13	Contains *espG2* and *espC*
EM3347	29M (IE6)	29.6	3341269..3370924	23	Contains *lifA*/*efa1*_IE6_, *nleE*_IE6_, *nleB*_IE6_, and *espL*_IE6_
EM3349	48M	27.4	4850071..4877448	37	
Total		586.7		770	

a–The Islands' nomenclature used by Iguchi *et al.* [2] is in brackets.

b–Based on Iguchi *et al.* [2].

### NleE_IE2_ is not required for IκB stabilization

**Figure 3:**
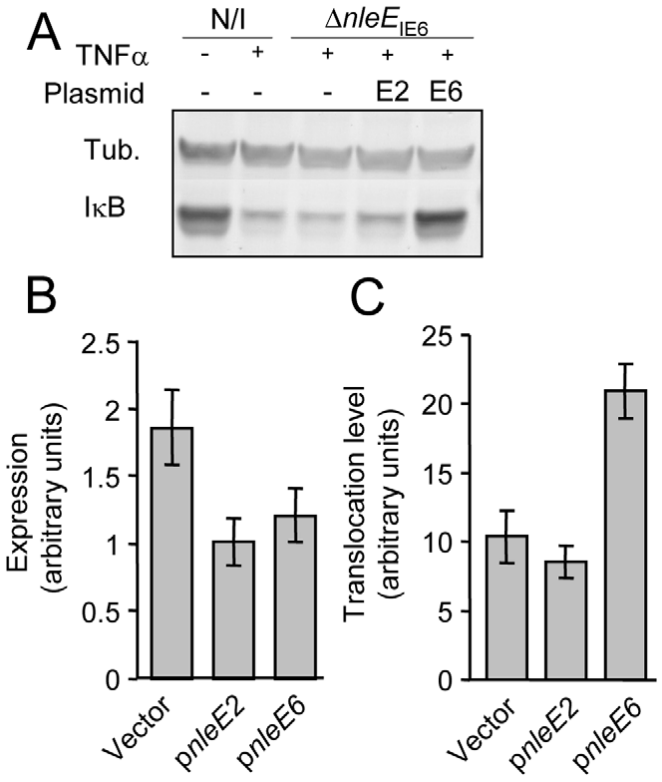
NleE of IE2 is deficient in translocation and inhibition of IκB degradation. (A) HeLa cells were either uninfected (N/I) or infected with a strain deleted of *nleE*_IE6_ (SC3680) (see Fig. 2A), complemented or not complemented with plasmids expressing NleE_IE2_ or NleE_IE6_ (indicated as E2 and E6, respectively). After 3h, cells were washed, treated with TNFα for 30 min and extracted. The extracts were analyzed by western blot with anti-IκB and anti-tubulin antibodies. (B and C) HeLa cells were infected with wild-type EPEC harboring plasmids expressing *nleE*_IE2_ or *nleE*_IE6_ fused to the *blaM* reporter gene (indicated as p*nleE2* and p*nleE6*, respectively). As a negative control, cells were infected with EPEC harboring the parental vector pCX341 (Vector). The β-lactamase activity in the infecting bacteria, reflecting the expression levels of the fusion proteins (B), and the β-lactamase activity in the infected HeLa cells, reflecting the levels of translocation of the fusion proteins into the HeLa cells (C) were determined. The experiment was done twice in triplicates and typical results are shown. Standard errors are indicated by bars.

EPEC encode two very similar *nleE* alleles. One allele, identified in our screen, is located in the IE6 region and the other is in the IE2 region [2]. We initially found that deletion of the IE6 region, but not of the IE2 region, caused deficiency in inhibition of IκB degradation (data not shown). However, the two proteins, NleE_IE2_ and NleE_IE6_, are identical, apart from an internal deletion of 56 residues in NleE_IE2_ (Fig. S1), and this similarity between the two proteins urged us to determine the activity of each of the two proteins. We first tested their ability to complement IκB destabilization in a strain deleted of *nleE*_IE6_. To this end, we expressed each of them on a plasmid carrying an identical promoter and ribosomal binding site. Results showed that only NleE_IE6_, but not NleE_IE2_, was able to attenuate IκB degradation (Fig. 3A). These results indicate that NleE_IE2_ is either not active in the host cell or is not translocated into the host cell. To differentiate between these two possibilities, we used the above mentioned plasmid where both proteins were fused to the β-lactamase translocation reporter protein, BlaM. The plasmids were introduced into EPEC and the ability to translocate them into infected cells was tested. We found that both NleE_IE2_-BlaM and NleE_IE6_-BlaM were expressed at similar levels in the bacteria (Fig. 3B). Importantly, however, only NleE_IE6_ was translocated into the host cell (Fig. 3C), suggesting that NleE_IE2_ is a cryptic effector. Cumulatively, these results define NleE_IE6_, but not NleE_IE2_, as the effector needed for inhibition of IκB degradation. Therefore, in this report the term “NleE” specifically refers to “NleE_IE6_”.

### NleE is required for complete inhibition of NF-κB translocation to the nucleus

To corroborate the notion that NleE is required for inhibition of NF-κB activation, we examined whether the *nleE* mutant is deficient in blocking the translocation of the p65 NF-κB subunit to the nucleus upon TNFα treatment. Because of the high similarity between the IE2 region and the IE6 region, we made the specific mutants (Δ*nleE*_IE6_, Δ*nleEB*_IE6_) in a strain deleted of its IE2 region (ΔIE2). Therefore, these specific strains were compared to their parental strain (indicated as WT_ΔIE2_ in the figures). It should be emphasized that the ΔIE2 mutant exhibited the wild-type phenotype in all the assays used in this study.

**Figure 4:**
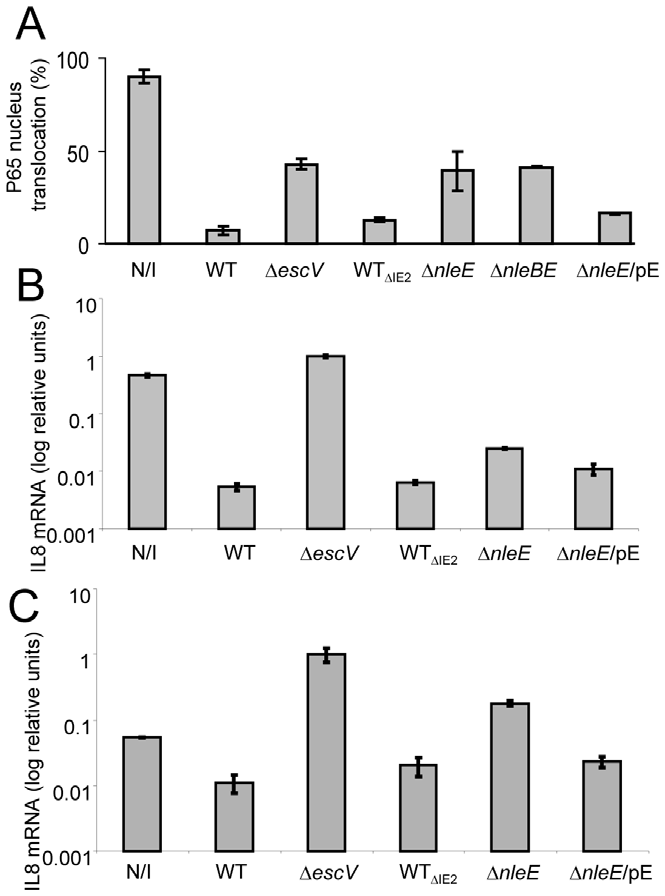
NleE is required to block IL-8 secretion by EPEC. AGS SIB02 cells expressing p65-GFP (A) or HeLa cells (B and C) were infected with the parental strain deleted of the IE2 region (strain EM3327, indicated as WT_ΔIE2_) or with several isognic mutants including a mutant deleted of *nleE* (strain SC3908, indicated as Δ*nleE*), a mutant deleted of nleBE (strain SC3909, indicated as Δ*nleBE*) and a mutant deleted of *nleE* and complemented with a plasmid expressing *nleE* (pSC3982), indicated as Δ*nleE*/pE. As positive and negative controls we used cells, which were either uninfected (N/I) or infected with wild type EPEC (WT) or with a TTSS mutant (strain SN1961, indicated as Δ*escV*). (A) After 3 h infection, the cells were washed, stimulated with TNFα for 30 min, fixed, and nuclei stained with Hoechst 33342. Nuclear translocation of p65-GFP was quantified by automated microscopy and image analysis software. NleE expression was induced with IPTG (0.01mM). Standard errors are indicated by bars (n = 200). (B and C) After 3 h infection, cells were washed and treated (B), or not (C), with TNFα to induce NF-κB and with gentamicin to kill the bacteria. After additional 3 h incubation the cells were harvested and RNA was extracted and analyzed for the amount of IL-8 transcripts. The amount of IL-8 mRNA in each strain is shown as a percentage of the level relatively to the transcript levels in the Δ*escV* mutant. The experiment was done twice in duplicates and typical results are shown. Bars indicate standard errors.

AGS SIB02 cells expressing p65-GFP were infected with the parental strain WT_ΔIE2_ or with the corresponding mutants: Δ*escV*, Δ*nleE*, Δ*nleBE*, and Δ*nleE* complemented with a plasmid expressing *nleE*. After 3 h, cells were TNFα-induced for 30 min, stained with Hoechst 33342 and analyzed by automated microscopy. The results show that whereas the wild type repressed ∼90% of the p65 translocation to the nucleus, the TTSS *escV* mutant was attenuated, exhibiting only ∼50% repression (Fig. 4A). These results indicate that EPEC inhibit p65 translocation by both TTSS-independent and TTSS-dependent pathways. Importantly, the *nleE* and *nleBE* mutants were as deficient as the *escV* mutant in blocking p65 translocation. Moreover, complementation of the *nleE* mutant with the wild-type *nleE* allele restored the bacteria's full capacity to inhibit the p65 translocation (Fig. 4A). These results support the hypothesis that the TTSS-mediated inhibition of p65 translocation is NleE-dependent. In addition, our results suggest that a putative TTSS-independent mechanism might function in parallel to NleE to inhibit p65 translocation.

### NleE is required for full inhibition of TNFα-induced IL-8 expression

To further substantiate our results, we used IL-8 expression as an additional read-out for NF-κB activation. Briefly, HeLa cells were infected with different EPEC strains or remained uninfected. Then, cells were washed and treated for 3 h with TNFα and gentamycin, to kill the remaining bacteria. RNA was then extracted from the cells and the amount of produced IL-8 mRNA was measured by real time PCR. In comparison to non infected cells or cells infected with EPEC *escV* mutant, both wild type and the ΔIE2 mutant exhibit a ∼100 fold repression of IL8 expression (Fig. 4B). In contrast, the *nleE* mutant exhibited a partial, less then 10 fold, repression of IL8 expression and this was moderately complemented by plasmid expressing native NleE (Fig. 4B). A more severe deficiency in repression of IL8 expression was exhibited by a double mutant *nleBE* (Fig. S3). Furthermore, a plasmid expressing *nleBE* restored IL8 repression to that seen in wild type EPEC (Fig. S3). Upon testing the amount of secreted IL8 protein instead of production of IL8 mRNA, similar results were obtained (Fig. S2). Taken together these results show that (i) NleE is required for full inhibition of IL-8 expression, (ii) NleB also contributes to this repression and iii) a putative TTSS effector(s), other then NleB and NleE might function in parallel to inhibit IL-8 expression.

### NleE is required to inhibit the EPEC-induced IL8 expression

The IL8 expression assay was found to be much more sensitive then testing translocation to the nucleus or the IkB degradation assay. This is probably since the latter are very transient events while the mRNA tends to accumulate, increasing the signal/noise ratio. Interestingly, using the IL8 expression assay we found that infection with the *escV* mutant was sufficient to induce IL8 expression in HeLa cells, albeit not as strong as that induced by TNFα (data not shown). This activation is possibly via the activity of flagellin, LPS or other PAMPs. We thus next asked whether NleE also inhibits the EPEC-induced IL8 expression. To this end we repeated the experiment described in Fig. 4B, but TNFα was omitted. We found that even the non infected cells produce certain levels of IL8 mRNA, but upon infection with EPEC *escV* mutant we observed a ∼10 fold increase in IL8 expression (Fig. 4C). In contrast, the EPEC wild type (or the ΔIE2 mutant) exhibited strong repression of the EPEC-induced IL8 expression. Importantly, the *nleE* mutant exhibited only a partial capacity to repress the self-induced IL8 expression. Similar results where observed when we used the double mutant *nleBE* instead of *nleE* mutant (Fig. S3). However, both the *nleE* or the *nleBE*, mutants were not as deficient in IL8 repression as the *escV* mutant (Fig. 4C and S3). Thus, we predict that additional putative effector might function in parallel to NleB and NleE to repress IL8 expression. In conclusion, our results clearly show that i) EPEC mediate a TTSS-dependent repression of self-induced IL8 expression; and ii) NleE is required for full repression of the EPEC-induced IL8 expression.

### NleE, expressed by HeLa cells, inhibits NF-κB translocation to the nucleus

**Figure 5:**
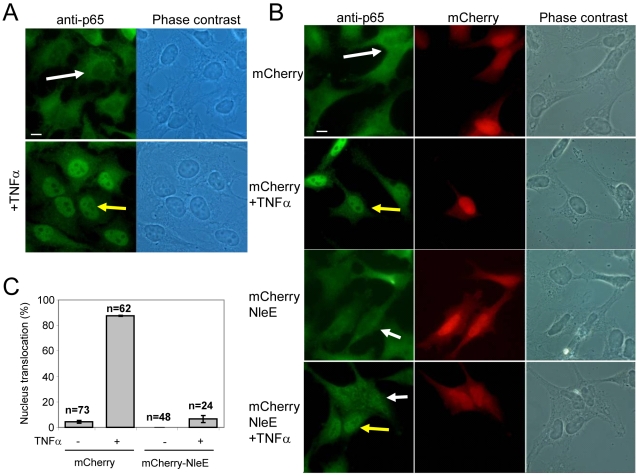
NleE is sufficient to block translocation of NF-κB to the nucleus. (A) HeLa cells were treated with TNFα for 1 h, or remained untreated, after which they were fixed and stained with anti-p65 (green) (bar represents 20 µm). (B) HeLa cells transfected with plasmid expressing mCherry or mCherry-NleE (red) were treated with TNFα for 1 hr, or remained untreated, after which they were fixed and stained with anti-p65 (green). Yellow arrows indicate cells exhibiting p65 translocation to the nucleus and white arrows indicate cells where the p65 remained cytoplasmic (bar represents 20 µm). (C) To quantify the results shown in (B), the percentage of red cells (expressing mCherry or mCherry-NleE) containing nuclear p65 was determined. The number of cells quantified is indicated and standard errors are indicated by bars.

We next examined whether NleE is sufficient for inhibition of NF-κB activation in the absence of the infecting bacteria and other putative effectors. To this end, we constructed a vector expressing mCherry fused to NleE (mCherry-NleE) and used it for transient transfection of HeLa cells. Untransfected cells (Fig. 5A) or cells transfected with either the mCherry-NleE vector or a vector expressing mCherry alone (Fig. 5B) were stimulated with TNFα for 1 h, or remained untreated. Next, these cells were fixed, stained with anti-p65 antibody, and analyzed by fluorescent microscopy to determine both the ability of the transiently expressed NleE to inhibit TNFα-induced migration of p65 to the nucleus and to determine its localization in the expressing cells. The TNF treatment induced strong migration of p65 to the nucleus in the untransfected cells (Fig. 5A), and in cells transfected with the negative control vector (Fig. 5B two upper panels and 5C). Importantly, the transiently expressed mCherry-NleE induced a strong inhibition of p65 translocation to the nucleus (Fig. 5B two lower panels and 5C). The expressed mCherry and mCherry-NleE were similarly distributed in the cells, predominantly in the cytoplasm (Fig. 5B). These results indicate that NleE_IE6_ is sufficient for inhibition of NF-κB migration to the nucleus presumably by IκB stabilization. Similar analysis using NleE_IE2_ instead of NleE_IE6_, show that NleE_IE2_ lost the ability to block p65 translocation to the nucleus (Fig. S4), highlighting the importance for NleE activity of the region between residues 49–115, which is deleted in NleE_IE2_ (Fig. S1).

### NleE inhibits phosphorylation of IκB

**Figure 6:**
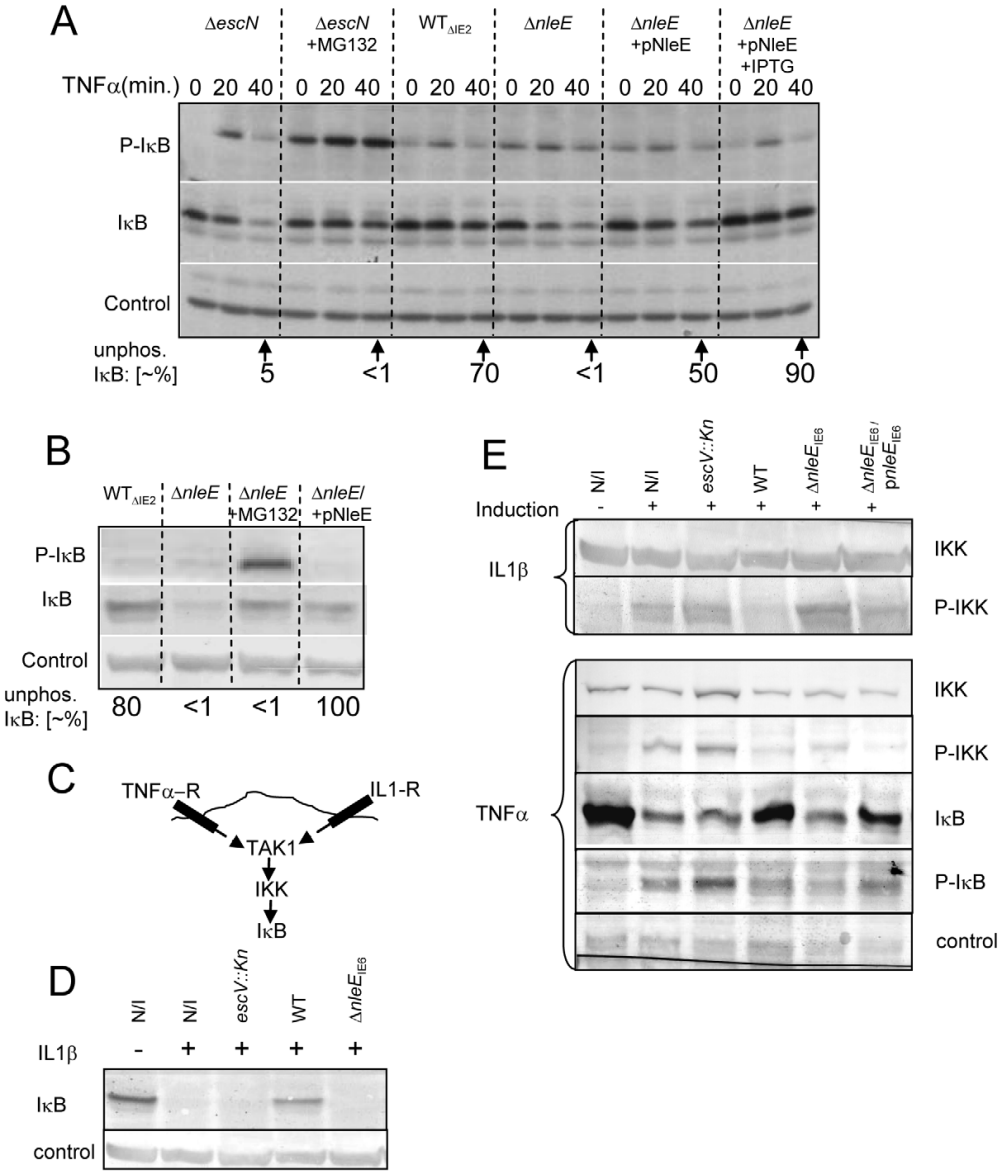
NleE inhibits IκB and IKKβ phosphorylation. (A, B) HeLa cells were infected for 3 h with different EPEC strains, as indicated followed by TNFα treatment in the absence or presence of proteasome inhibitor (MG132) or IPTG (0.01 mM) as indicated. Proteins were extracted at 0, 20, and 40 min post TNFα treatment. The blots were developed with anti-IκB (IκB), anti-phospho IκB (P-IκB), or anti-tubulin antibodies (loading control). The strains used are indicated above the lanes. The parental strain deleted of the IE2 region, strain EM3327 is indicated as WT_ΔIE2_. The band-densities of IκB and P-IκB at 40 min post TNFα treatment were measured and the relative amounts of the unphosphorylated IκB were calculated (indicated as “unphos. IκB [∼%]”) and shown below the corresponding lanes. (B) HeLa cells were infected as in (A) and proteins were extracted at 40 min post TNFα treatment. The blots were developed and the ∼% unphos. IκB was determined as in (A). (C) Schematic of the signaling pathways initiated upon activation of the TNF and IL1 receptors (TNF-R and IL1-R, respectively). (D and E) HeLa cells were infected with different strains as indicated or not (N/I) and treated with TNFα or IL1β as indicated. Proteins were then extracted and the levels of IκB (D and E), phospho-IκB (P-IκB), IKKβ and phospho-IKKβ (P-IKK) (E), were determined by immunoblot analysis with anti-IκB, anti-IKKβ and anti-phospho-IKK respectively.

Different NF-κB activating pathways converge at the level of IKK phosphorylation, which subsequently leads to IκB phosphorylation, targeting it to ubiquitination and proteasome-mediated degradation [6]. We thus tested whether NleE inhibits the TNFα-induced IκB phosphorylation. Cells were infected with different EPEC strains followed by TNFα treatment. The levels of IκB and phospho-IκB were then determined by immunoblot analysis with the appropriate antibodies and the relative accumulation of unphosphorylated IκB was determined. For a negative control, we used cells infected with the *escN* mutant, which cannot stabilize IκB (Fig. 1A). Indeed, in cells infected with this mutant we noted increased IκB phosphorylation followed by its degradation. However, the addition of proteasome inhibitor (MG132) resulted in accumulation of phosphorylated IκB (Fig. 6A). As a parental strain, we used the ΔIE2 strain (WT_ΔIE2_), which, like wild-type EPEC, efficiently protected IκB from degradation (Fig. 1A and 6A). Importantly, the accumulated IκB in these cells was mostly unphosphorylated. In contrast, the corresponding *nleE* mutant failed to induce accumulation of unphosphorylated IκB, exhibiting a phenotype similar to that of the *escN* mutant (Fig. 6A). Taken together, these results indicate that wild-type EPEC stabilizes IκB by preventing its phosphorylation and that NleE is required for this activity. Indeed, complementing the Δ*nleE* mutant with a plasmid expressing NleE restored the bacteria's capacity to induce the accumulation of unphosphorylated IκB (Fig. 6A).

These results suggest that one can restore the inability of the Δ*nleE* mutant to prevent IκB degradation by two alternative approaches: (i) by treatment with proteosome inhibition, to inhibit phospho-IκB degradation, or (ii) by complementation with a plasmid expressing *nleE*, to block IκB phosphorylation. To compare the efficiency of these two treatments, we infected HeLa cells with the ΔIE2 strain (WT_ΔIE2_), Δ*nleE* mutant, or with the Δ*nleE* mutant complemented either by proteosome inhibitor (MG132) treatment upon TNFα induction, or by a plasmid expressing *nleE*. The results show that both treatments similarly stabilized the IκB. However, the first treatment led to a strong phosphorylation of the accumulated IκB whereas when NleE was added, the accumulated IκB remained unphosphorylated (Fig. 6B). These results further support the notion that NleE stabilizes IκB by inhibiting its phosphorylation.

### NleE inhibits IKKβ activation induced by TNFα or IL1β

The signaling pathways induced by the TNF receptor (TNFR) is different from that induced by the IL1 or TLR receptors, but both converge at the level of IKK activation by TAK1 (Fig. 6C, [16]). The inhibition of the self-induced IL8 expression by NleE (Fig. 4C), is hinting that NleE functions downstream to the pathways converging point. To directly test this prediction we tested whether EPEC is capable of inhibiting IL1β-induced degradation of IκB. Importantly, we found that wild type EPEC, but not the *nleE* mutant, inhibited the IL1β-induced IκB degradation (Fig. 6D). These results confirmed that NleE functions downstream to the signaling converging point. We next tested whether NleE can block the phosphorylation and thus activation of IKKβ. To this end we extracted proteins from cells, which were infected with different strains and then treated with TNFα or IL1β as indicated (Fig. 6E). The extracted proteins were subjected to western analysis using anti-IKKβ, anti-phospho-IKK, anti-IκB and anti-phospho-IκB antibodies. The results show that, treatment with either TNFα or IL1β induced IκB and IKK phosphorylation in non infected cells or cells infected with the *escV* mutant. We also found that wild type EPEC, but not the *nleE* mutant, inhibited this IKK phosphorylation. The same inhibition is noted for the IkB phosphorylation, in the wildtype strain, However, due to IkB degradation, less protein is noted and thus its phosphorylation cannot be seen (Fig. 6E). Complementation with plasmid expressing wild type *nleE* allele only partially, but consistently restored the inhibition of IKKβ phosphorylation (Fig. 6E). These results suggest that NleE block activation of IKKβ. Taken together our results indicate that NleE blocks the NF-κB signaling cascade downstream to the converging point of the TNFα and IL1β signaling pathways, but upstream to IκB phosphorylation, possibly by direct blocking TAK1 or IKKβ activation.

## Discussion

In this report we showed that NleE of EPEC stabilizes the NF-κB inhibitor, IκB, via inhibition of its phosphorylation, thereby preventing NF-κB signaling. This activity of NleE was discovered using an unbiased screen of EPEC strains deleted of most of the EPEC-specific genes. We showed that an *nleE* mutant is deficient in blocking IκB phosphorylation and in preventing its degradation. Moreover, an *nleE* mutant was attenuated in blocking TNFα-induced NF-κB migration to the nucleus as well as in IL-8 expression and secretion. These abilities were restored to the mutant upon complementation with a plasmid expressing the wild-type *nleE* allele. Importantly, we showed that NleE expressed in HeLa cells blocks NF-κB translocation to the nucleus upon TNFα treatment. Taken together, our findings indicate that NleE is sufficient to inhibit NF-κB signaling by blocking IκB phosphorylation. Further analysis suggest that NleE blocks the NF-κB signaling cascade downstream to the converging point of the TNFα and IL1β signaling pathways, but upstream to IκB phosphorylation, possibly by directly blocking TAK1 or IKKβ activation.

We also show that NleB enhances NleE activity. The *nleE* mutant still showed residual inhibition of IκB degradation, which was eliminated upon further deletion of *nleB*. Moreover, complementation of the *nleBE* double mutant with a plasmid expressing *nleBE* was more efficient than a plasmid expressing only *nleE*. These results suggest that NleB plays a role in IκB stabilization. Similar results were obtained when expression of IL8 was used as a readout for inhibition of the NF-κB signaling. The mechanism underlying NleB function is not yet apparent. Nevertheless, the notion that NleB and NleE function together is supported by the facts that *nleE* form a putative bicistronic operon with *nleB* and that *nleE* is consistently associated with *nleB* in natural isolates of diarrheagenic EPEC [17]. Other isolates of EPEC, EHEC, and *C. rodentium* carry *nleB* and *nleE* alleles almost identical to *nleBE*_IE6_ investigated in this study (Supplemental material Fig. S1). We predict that all NleBE proteins function similarly.

**Figure 7:**
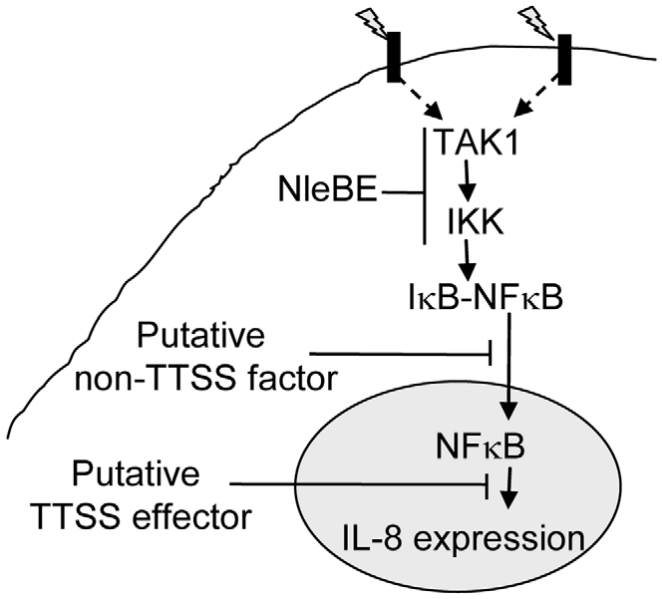
Schematic of inhibition of NF-κB signaling by EPEC. Signals (indicated as lightning) sensed by different receptors elicit signal transduction cascades that converge at the level of TAK1 activation and IKK phosphorylation, which subsequently leads to IκB phosphorylation. This step is inhibited by NleBE. NleE inhibits IKKβ phosphorylation, but the exact target of NleE and NleB is yet to be defined. The phosphorylated IκB is subjected to ubiquitination and proteasome-mediated degradation, allowing NF-κB translocation to the nucleus and activation of expression of target genes including IL-8. Our results predicate that in addition to NleBE, a putative non-TTSS factor and a putative TTSS effector might inhibit NF-κB translocation to the nucleus and IL-8 expression, respectively (Fig. 4).

The phenomenon of effectors functioning in parallel is common in EPEC [1]. Interestingly, the TTSS mutants (*escV*) and Δ*nleBE* were only partially deficient in blocking the TNFα-induced migration of NF-κB to the nucleus, suggesting that an additional TTSS-independent mechanism might function in parallel to NleBE to inhibit translocation of NF-κB to the nucleus (Fig. 7). This activity might be related to damping of the signal due to continuous exposure to PAMPs like flagellin or LPS. Interestingly, the TTSS *escV* mutant was completely deficient in inhibition of IL8 expression, while the *nleBE* mutant was only partially deficient in this function. We therefore predict that EPEC encode additional putative effector(s) that function in parallel to NleBE by blocking IL-8 expression (Fig. 7).

Where as in this study we showed that NleE represses NF-κB activation, a recent report suggested exactly the opposite, i.e. NleE and OspZ, an NleE-homolog encoded by *Shigella*, activates NF-κB signaling [18]. The discrepancy between the two studies might result from the use of different cell lines. In their study, Zurawski *et al.* [18] used cell lines that activate NF-κB upon sensing of either TNFα or bacterial PAMPs including LPS. The latter might complicate the interpretation of the results. To avoid such complications, we used in this study HeLa and AGS cells, which are inert to LPS. Moreover, we adjusted the experimental conditions used in this study, so that the NF-κB activation by other bacterial PAMPs was insignificant. This facilitated the uncoupling of effector injection and NF-κB activation, which occurred only upon addition of TNFα. An alternative, although less likely explanation for the inconsistency between the two studies is that NleE functions differently in different types of cell or different cell lines.

Like EPEC, *Yersinia* also employs an injected effector, YopJ, to block IκB phosphorylation. YopJ is an acetyl transferase that acetylates critical IKKβ residues and thus prevents its activation [19]. Although both NleE and YopJ block IKKβ phosphorylation, they are very different in sequence, which probably reflects functional differences. We are currently investigating the NleE's mode of action. Other effectors that interfere with NF-κB function include the *Salmonella* SspH, and *Shigella* IpaH9.8, which are targeted to the host cell nucleus and inhibit NF-κB-dependent transcription [20]. *Shigella* also uses OspG, which inhibits ubiquitination of phospho-IκB [15]. Interestingly, EPEC encode an OspG homolog, NleH. However, we found that an EPEC strain, mutated in its two *nleH* alleles, still inhibit IκB degradation. Yet, our analysis indicates that additional TTSS effector(s) inhibits NF-κB signaling (Fig. 7) and we can not exclude the possibility that this putative effector is *nleH*.

The role of NleE in EPEC virulence was not tested, since an animal model is not yet available, but it was tested with *C. rodentium*. Importantly, NleE was found to be required for full virulence of *C. rodentium* upon infection of wild-type mice, but this requirement was diminished upon infection of mice deficient in TLR4 [21],[22]. Our results revealed the rationale behind this intriguing phenomenon. We argue that NleE-mediated NF-κB repression is no longer needed if the host itself is deficient in TLR4/LPS-induced NF-κB signaling.

In conclusion, we show that NleE blocks IκB phosphorylation by IKK and thus it inhibits NF-κB signaling. We also show that NleB enhances NleE's activity and that EPEC probably use additional mechanisms to interfere with other constituents of the NF-κB signaling pathway. This is presumed to have multiple consequences on the course of EPEC infection and the maturation of both innate and adaptive host immune response.

## Materials and Methods

### Bacterial strains, plasmids, and primers

Bacterial strains, plasmids, and primers used in this study are listed in Table S1, S2, and S3, respectively (Supplemental material). Deletions in the EPEC chromosome were constructed using the primers listed in Table S3, as described [23]. For bacterial expression, the genes were cloned in the pSA10 expression vector as described [24]. In most cases the leakiness of the Tac promotor was enough for gene expression. When indicated, IPTG (0.01mM) was added. For the cloning procedure, genes were amplified by RCR using the primers listed in Table S3. Formation of plasmid-borne *nleE*-*blaM* fusions were carried out as described [25] using the plasmid pCX341 and primers as listed in Table S3. The *nleE*_IE2_ DNA was amplified from EPEC ΔIE6 mutant and that of *nleE*_IE6_ from EPEC ΔIE2 mutant. For formation of mCherry fusions, the EGFP gene of pEGFP-N1 (Clonetech) was excised from the plasmid using the *Not*I and *Bam*HI and replaced by *mCherry* taken from pREST-mCherry, resulting in pMS2841. This plasmid was transformed into pSC4141 by introduction of a unique *scaI* site at the mCherry 3′, eliminating its stop codon in the process. This was done using QuikChange site-directed mutagenesis kit (Stratagene #200518-5) and primers listed at Table S3. This plasmid was further used to create the transcriptional fusion: mCherry-*nleE*_IE6_ *-6his* (pSC4144) and mCherry-*nleE*_IE2_ *-6his* (pSC4350), using primers listed at Table S3.

### Analysis of IκB stability and IκB and IKKβ phosphorylation

HeLa cells (9×10^5^) in 4 cm plates were infected with a 1∶100 dilution in DMEM of bacteria grown overnight statically at 37°C (multiplicity of infection, MOI∼1∶100). Following 3 h infection in 5% CO2, at 37°C, the medium was replaced with fresh DMEM with or without either 10 ng/ml TNFα for 40 min (or 20 ng/ml IL-1β for 20 mins). When indicated, the infecting bacteria were supplemented with 0.01 mM IPTG at 1.5 h post inoculation. To terminate the infection, cells were washed with 3 ml of cold TBS (20 mM Tris-HCl, pH 7.4, 150 mM NaCl), scraped with 1 ml of cold TBS, collected and centrifuged, (800g, 2 min at 4°C). The pellet was resuspended in 40 µl lysis buffer (0.5% Triton-×100, 20mM Tris-HCl pH 7.2, 0.2 mM VO_4_, 10 mM NaF, 30 µl of complete inhibitor Roche) and centrifuged (20,000g, 3 min at 4°C). Supernatant was either transferred for protein quantification assay (BCA assay) or to a tube with loading dye (LDS sample buffer, NuPAGE), boiled for 10 min, and then centrifuged (20,000g, 3 min). Samples were quantified using bicinchoninic acid (BCA) and copper sulfate. Equal protein concentration for each sample was then loaded on SDS-PAGE gel, transferred to PVDF membrane, and reacted with antibodies against IκB (1∶1000), Tubulin, as a loading verification control (1∶2500), or phospho-IκB (1∶1000, Cell Signaling). When indicated, 20 mM MG132 (1∶1000) was used. Protein band density was quantified using Tina software (version 2.09) and the percentage of the unphosphorylated IκB was determined by calculating the relative phosphorylated IκB out of the total IκB shown for each lane. IKKβ analysis was done as described for IκB except that induction time with TNFα was reduced to 10 min and with IL1β it remained 20 min. IKK detection was preformed by western blot analysis using anti-IKKβ antibody (1∶1000, Cell Signaling Technology, #2684) and Phospho-IKKα (Ser180)/IKKβ (Ser181) antibody (1∶1000, Cell Signaling Technology, #2681S)

### Analysis of p65 translocation to the nuclei

Generation of the p65-GFP-expressing cell line and the automated image analysis to quantify translocation of p65-GFP is described elsewhere (Bartfeld *et al.*, submitted). Briefly, SIB02 cells are AGS cells lentivirally transduced to express p65-GFP. SIB02 cells, seeded in 96-well-plates, were inoculated with EPEC strains at MOI 1∶100, incubated for 3 h and subsequently activated by 10 ng/ml TNFα. After an appropriate incubation time, cells were fixed using 100% ice-cold methanol and stained with Hoechst 33342 (2 µg/ml). Images of ∼200 cells were acquired using automated microscopy (Scan^∧^R, Olympus) and translocation of p65-GFP to the nucleus was subsequently quantified using Scan^∧^R image analysis software (Olympus) as described (Bartfeld *et. al.*, submitted). Cells with nuclear p65-GFP above the defined threshold were termed “active” and the percentage of active cells per well was calculated.

### Nuclear cytoplasmic fractionation

HeLa cells (2.8×10^6^) were seeded in 10 cm plates. The next day, the cells were infected with EPEC for 3.5 h as described. Cells were then washed, treated with 20 ng/ml TNFα in DMEM for 30 min., washed with cold PBS, scrapped, transferred to Eppendorf tubes and centrifuged (5 mins, 660 g, 4°C). Then, the pellet was resuspended in 7 times the volume of Hypotonic Lysis Buffer (HLB, 10mM HEPES pH 7.6, 0.1mM EDTA, 0.1 mM EGTA, 2mM DTT, 10mM KCl, 1mM PMSF, 0.75mM Sperimidine, 0.15mM Sperimide, 20mM PNPP, 1µM okadaic acid and 5µg/ml protease inhibitor), incubated on ice for 15 mins and then 0.2% NP40 was added gently following gentle mixing for several minutes. The lysate was then centrifuged (5 mins, 2600 g, 4°C), the supernatant (cytoplasmic fraction) was recovered and the pellet (nuclear fraction) was washed with HLB once and then resuspended in 100 µl Nuclear Extraction Buffer (NEB, 210 mM HEPES pH 7.6, 0.2 mM EDTA, 2 mM EGTA, 0.5 mM DTT, 25% Glycerol, 0.42 M NaCl, 20 mM glycerophosphate, 29 mM PNPP, 1 µM okadiac acid, 1 mM NaVO_4_, 5 µg/ml protease inhibitor, 0.75 mM Sperimidine, 0.15 mM Sperimide). The nuclear lysets were then vortexed, mixed vigorously (1400 rpm, 30 min., 4°C) and clarified (20,000 g, 10 min, 4°C). Protein concentrations were determined (BCA kit, Sigma), adjusted and the extracts were used for western analysis using anti-NF-κB p65 antibodies (Santa Cruz, SC372). The quality of the fractionation was confirmed using tubulin as a cytoplasmic marker and fibrillarin as a nuclear marker.

### Expression and translocation of NleE-BlaM fusions

To determine translocation levels, overnight cultures of wild-type EPEC containing plasmids expressing NleE-BlaM were diluted 1∶50 in DMEM and used to infect HeLa cells for 3 h. Cells were then washed and stained with CCF2 for 2.5 h as described [26], washed in cDMEM, excited at 405 nm, and then emission at 465 nm and 535 nm was recorded (SPECTRAFluor, TECAN). The amount of translocation was determined as described [26]. As a negative control, we used EPEC expressing unfused BlaM (Vector). To determine expression levels, the unattached bacteria were harvested, washed, and lysed by repeated freezing and thawing in PBS containing 1 mM EDTA, 1 mg/ml lysozyme, and 0.1% Triton-×100. The BlaM activity in the lysate was determined using nitrocefin as substrate and the rate of product accumulation per number of bacteria (OD 600) was determined as described [25].

### IL-8 expression

HeLa cells (7×10^5^) in 6 wells plates were inoculated with a 1∶100 dilution in DMEM of bacteria grown overnight statically at 37°C (multiplicity of infection, MOI∼1∶100) and incubated for 3 h (5% CO_2_, 37°C). To terminate the infection and induce IL8 expression, the medium was replaced with fresh DMEM supplemented with 2% FCS, 100ug/ul gentamicin and with or without 10 ng/ml TNFα and incubated for additional 3 h. Cells were than washed with 2 ml of cold TBS (20 mM Tris-HCl, pH 7.4, 150 mM NaCl), scraped with 1 ml of cold TBS, collected and centrifuged, (800 g, 2 min, 4°C). RNA was extracted using the MasterPure Complete DNA and RNA Purification Kit (EPICENTRE Biotechnologies) and used to synthesize cDNA with the Verso cDNA kit (Thermo scientific). hHPRT transcript levels were used to normalize total RNA levels in samples. Real time analysis was than conducted using Absolute Blue QPCR SYBR Green (Thermo scientific) in a real-time cycler (Rotor-Gene 6000, Corbett).

### Transfection of pCherry-*nleE* into HeLa cells and p65 staining

HeLa cells were transfected using ExGen500 (Fermentas), as recommended by the manufacturer, with 1 µg of pMS2841 (pmCherry), pSC4144 or pSC4350 (pmCherry*-nleE*s) or were not transfected. After 24 h, the medium was replaced with fresh DMEM containing, or not containing, 10 ng/ml TNFα. After 1 h, cells were fixed (3.7% PFA in PBS for 10 min and washed with PBS), perforated (with 0.25% Triton-X100 in PBS for 10 min and washed twice with PBS) and blocked (2% BSA in TBS) at 4°C for 16 h. Cells were then stained using anti-p65 (SC109, Cell Signaling) antibodies (1∶300 in TBS) overnight and further stained with CY-488 goat anti-rabbit (Cell Signaling) (1∶1000 in TBS) for 1 h. Slides were analyzed by fluorescent microscopy.

## Supporting Information

Table S1 Strains. All strains are made in EPEC E2348/69.
(0.02 MB PDF)

Table S2 Plasmids
(0.02 MB PDF)

Table S3 Primers. F = forward, R = reverse.
(0.02 MB PDF)

Figure S1 Comparison between different NleE genes of different EPEC and EHEC strains. The E2348 NleE_IE6_ is indicated as E2348-NLEE2 and NleE_IE2_ as E2348-NLEE1). Other NleE proteins are those of two EPEC strains (O111 B171 and O103 E22), two EHEC O157 strains (Sakai and EDL933) and *Citrobacter rodentium*.
(1.58 MB TIF)

Figure S2 NleE is involved in blocking IL8 secretion. HeLa cells (8×10^4^ per well, seeded in 24-well plate) were infected for 3h with different EPEC strains as indicated or remained uninfected (N/I). After 3.5 h, supernatants were replaced with 300 ul DMEM, 2% FCS, and 50 µg/ml gentamycin with or without 10 ng/ml TNFα. After 16 h, 100 µl of cleared supernatant taken from each well was used for IL-8 measurements using Human CXCL8/IL-8 Quantikine immunoassay assay (R & D), according to the manufacturer's instructions. PBS and IL-8 were used as negative and positive controls for the detection assay. The relative amounts of IL-8 are shown. The experiment was done twice in duplicates and typical results are shown. Standard errors are indicated by bars. In the case of Δ*escV* (indicated by a vanishing colored bar), the signal was above the upper limit of the detection levels. Untreated and uninfected cells secreted ∼300 units of IL-8, whereas TNFα treatment induced a 10-fold increase in IL-8 secretion (∼3000 units) (Fig. 4B). In contrast, pre-infection with wild-type EPEC or with the ΔIE2 mutant (WT_ΔIE2_), reduced IL-8 secretion below the detection levels, even upon TNFα treatment. Furthermore, the TTSS *escV* mutant was completely deficient in blocking IL-8 secretion. In conclusion, EPEC strongly reduces IL-8 secretion by a TTSS-dependent mechanism. Importantly, we found that the Δ*nleE* or Δ*nleEB* mutants were strongly deficient in blocking IL-8 secretion but not as deficient as the TTSS mutant (*escV*). These results show that (i) NleE is required for full inhibition of IL-8 secretion and (ii) other putative TTSS effector(s) might function in parallel to NleE to inhibit IL-8 expression and/or secretion.
(1.62 MB TIF)

Figure S3 A mutant baring a deletion of *nleBE* is deficient in repressing self-activated, or TNFα-induced, IL8 repression. HeLa cells were infected with the indicated strains and analyzed as described in Fig. 4B and 4C. To terminate the infection and induce IL8 expression, the medium was replaced with fresh DMEM supplemented with 2% FCS, 100ug/ul gentamicin and with or without 10 ng/ml TNFα and incubated for additional 3 h. Cells were than washed with 2 ml of cold TBS (20 mM Tris-HCl, pH 7.4, 150 mM NaCl), scraped with 1 ml of cold TBS, collected and centrifuged, (800 g, 2 min, 4°C). RNA was extracted using the MasterPure Complete DNA and RNA Purification Kit (EPICENTRE Biotechnologies) and used to synthesize cDNA with the Verso cDNA kit (Thermo Scientific). hHPRT transcript levels were used to normalize total RNA levels in samples. Real time analysis was than conducted using Absolute Blue QPCR SYBR Green (Thermo Scientific) in a real-time cycler (Rotor-Gene 6000, Corbett). The amount of IL-8 mRNA in each strain is shown as a percentage of the level relatively to the transcript levels in the Δ*escV* mutant. The experiment was done twice in duplicates and typical results are shown. Bars indicate standard errors.
(0.44 MB TIF)

Figure S4 NleE_IE2_ is deficient in blocking TNF-induced translocation of p65 to the nucleus. HeLa cells were transected with plasmids expressing mCherry, mCherry-NleE_IE2_ or mCherry-NleE_IE6_. The expressing cells were treated with TNFα for 1 h, or remained untreated, after which they were fixed and stained with anti-p65. The slides were analyzed by fluorescent microscopy and the percentage of red cells (expressing mCherry or mCherry fused to NleE_IE6_ or NleE_IE2_) containing nuclear p65 was determined. The number of cells quantified is indicated and standard errors are indicated by bars. The results show that while NleE_IE6_ inhibited p65 translocation, NleE_IE2_ failed to do so.
(2.09 MB TIF)
