# Supplementary material for: The Type III Secretion Effector NleE Inhibits NF-κB Activation
Source: PLoS Pathog. 2010 Jan 29;6(1):e1000743. doi: 10.1371/journal.ppat.1000743 (PMC2813277; doi:10.1371/journal.ppat.1000743)
Supplement: Table S1 — Strains. All strains are made in EPEC E2348/69. (0.02 MB PDF) [file ppat.1000743.s001.pdf]

**Name**	**Description**	**Primers**	**markers**	**source**
E2348/69	Wild type			
CVD452	Δ*escN::kan*		Kan^R	[1]
SN1961	Δ*escV::kan*		Kan^R	[2]
CN2695	Δ*nleH*$_{PP2}$*::pCN2691*		Amp^R, Sm^R	This study
CN2785	Δ*nleH*$_{PP6}$*::kan*	#435 + #436	Kan^R	This study
CN2815	Δ*nleH*$_{PP6}$*::kan*, Δ*nleH*$_{PP2}$*::pCN2691*		Kan^R, Amp^R	This study
EM3327	ΔIE2::*kan*	#668 + #669	Kan^R	This study
EM3347	ΔIE6::*kan*	#677 + #678	Kan^R	This study
SC3518	Δ*orf1, EspL, nleBE, orf2*::*kan*	#748 + #749	Kan^R	This study
SC3678	Δ*orf2::kan*	#888 + #889	Kan^R	This study
SC3680	Δ*nleE, orf2::kan*	#884 + #889	Kan^R	This study
SC3681	Δ*nleBE, orf2::kan*	#882 + #889	Kan^R	This study
SC3720	Δ*espL, nleBE, orf2::kan*	#861 + #889	Kan^R	This study
SC3722	Δ*orf1, EspL, nleBE, orf2*::*kan*	#880 + #889	Kan^R	This study
SC3908	ΔIE2*::kan* and *nleE* $_{IE6}$*::tet*	#948 + #949	Kan^R, Tet^R	This study
SC3909	ΔIE2*::kan* and *nleBE* $_{IE6}$*::tet*	#946 + #949	Kan^R, Tet^R	This study
SC4102	ΔIE2*::cm* and *nleB* $_{IE6}$*::tet*	#946 + #947	Cm^R, Tet^R	This study

References:

1. Jarvis KG, Giron JA, Jerse AE, McDaniel TK, Donnenberg MS, et al. (1995) Enteropathogenic Escherichia coli contains a putative type III secretion system necessary for the export of proteins involved in attaching and effacing lesion formation. Proc Natl Acad Sci U S A 92: 7996-8000.
2. Nadler C, Shifrin Y, Nov S, Kobi S, Rosenshine I (2006) Characterization of enteropathogenic Escherichia coli mutants that fail to disrupt host cell spreading and attachment to substratum. Infect Immun 74: 839-849.
