# Supplementary material for: The Type III Secretion Effector NleE Inhibits NF-κB Activation
Source: PLoS Pathog. 2010 Jan 29;6(1):e1000743. doi: 10.1371/journal.ppat.1000743 (PMC2813277; doi:10.1371/journal.ppat.1000743)
Supplement: Table S2 — Plasmids (0.02 MB PDF) [file ppat.1000743.s002.pdf]

**Name**	Description	**Primers**	**Remarks**	**source**
pCN2691	pGP704 containing 425bps fragment of *nleH*$_{PP2}$	F398, R399	Amp^R	This study
pSA10	Expression vector pKK177-3 derivative		Amp^R	[1]
pSC3753	pSA10 containing *nleE*$_{IE6}$-6His, using *EcoR*I and *Pst*I, PCR on EM3327	F875, R876	Amp^R	This study
pSC4104	pSA10 containing *nleBE* $_{IE6}$-6His with its promoter region, using *EcoR*I and *Pst*I, PCR on EM3327	F941, R876	Amp^R	This study
pSC4103	pSA10 containing *nleB* $_{IE6}$-6his with its promoter region, using *EcoRI* and *PstI*, PCR on EM3327	F941, R874	Amp^R	This study
pCN2691	pGP704 containing 425bps fragment of *nleH*$_{PP2}$	F398, R399	Amp^R	This study
pCX341	Vector for formation of fusions with the *blaM* reporter		Tet^R	[2]
pGH3760	pCX341 containing *nleE*$_{IE2}$, EcoRI and KpnI, PCR on EM3347	F807, R808	Tet^R	This study
pGH3761	pCX341 containing *nleE*$_{IE6}$, EcoRI and KpnI, PCR on EM3327	F807, R811	Tet^R	This study
pMS2841	The EGFP gene of pEGFP-N1 (Clonetech) was replaced by *mCherry* from pREST-mCherry inserted into *Not*I and *Bam*HI sites	F528 , R529	Kan^R	This study
pSC4141	A plasmid expressing *mCherry* deleted of its stop codon and with scaI site added	F982, R983	Kan^R	This study
pSC4144	A plasmid expressing *mCherry* fused to *nleE* $_{IE6}$*-6his* via CMV promoter (derivative of pEGFP, Clonetech), PCR on strain EM3327	F984, R985	Kan^R	This study
pSC4350	A plasmid expressing *mCherry* fused to *nleE* $_{IE2}$*-6his* via CMV promoter (derivative of pEGFP, Clonetech), PCR on strain EM3347	F984, R985	Kan^R	This study

References:

1. Schlosser-Silverman E, Elgrably-Weiss M, Rosenshine I, Kohen R, Altuvia S (2000) Characterization of Escherichia coli DNA lesions generated within J774 macrophages. J Bacteriol 182: 5225-5230.
2. Mills E, Baruch K, Charpentier X, Kobi S, Rosenshine I (2008) Real-time analysis of effector translocation by the type III secretion system of enteropathogenic Escherichia coli. Cell Host Microbe 3: 104-113.
