# Supplementary material for: The Type III Secretion Effector NleE Inhibits NF-κB Activation
Source: PLoS Pathog. 2010 Jan 29;6(1):e1000743. doi: 10.1371/journal.ppat.1000743 (PMC2813277; doi:10.1371/journal.ppat.1000743)
Supplement: Table S3 — Primers. F = forward, R = reverse. (0.02 MB PDF) [file ppat.1000743.s003.pdf]

Name	Sequence	Used to create:
Primers used for the creation of large EPEC chromosomal island deletions.<br>Letters in bold for antibiotics cassette homology.		
F695	CTTATCAATTTGTTGCAACGAACAGGTCACTATCAGTCAAAATAAAATCAT**GTGTAGGCTGGAGCTGCTTC**	EM3321
R696	GTTCATTTTCACTCTCTGGCGAGTGCCTCGATTACTGCGATATTTAGTTAA**CATATGAATATCCTCCTTAG**	EM3321
F665	CGCTATCACGCGAGGAGATTTTGAGGATGGTTGAGAGGATTGCTGGGTAGT**GTGTAGGCTGGAGCTGCTTC**	EM3323
R666	TGACGCTGGCAGGGACAATCATCGTCATGGTATGGCCAGGATTAGGATAAA**CATATGAATATCCTCCTTAG**	EM3323
F628	TTAATTATCAGTAAATTAATGTAAGTAGGTCATTATTAGTCAAAATAAAA**GTGTAGGCTGGAGCTGCTTC**	EM3325
R629	TGTGTGCCTCCGTTTGCCCCACCAATATATATTCAGCACAAGAAACACAG**CATATGAATATCCTCCTTAG**	EM3325
F668	GATTGCGTACGTTCGGCACTTTGCCAACGTACGCTACATGCATACAGTTAA**GTGTAGGCTGGAGCTGCTTC**	EM3327
R669	CCAGCCCTGTGCAGGCTGGCGTCAGAGGAATGAAGCCTGGTGAGTGAGCTT**CATATGAATATCCTCCTTAG**	EM3327
F698	TTACAACACTTTTTTCCTGAGATGGTGACGGATATTCCATATGGTGTTTAT**GTGTAGGCTGGAGCTGCTTC**	EM3329
R699	TGGCGCGCTTCTCGTCTGCTGAATAAGAAGGTGAGCGAATTAGATGATTAA**CATATGAATATCCTCCTTAG**	EM3329
F701	GTACGACTGGATCCGTGGGTGTTTATGTTCTCCGCAGGATATCGTTTTTAA**GTGTAGGCTGGAGCTGCTTC**	EM3331
R702	GCGGCACTGAAAGCTTTTGTACAACCGATGAAAGCGGCTACACGACGTTAA**CATATGAATATCCTCCTTAG**	EM3331
F704	AAAAACACAGCCAACCACAACAAATAACAGGGATGTGGTCACTTTGTGGAT**GTGTAGGCTGGAGCTGCTTC**	EM3333
R705	CAGAAGTTAAAAAGCCGGGTCGAAAACGCTTCGCCCAAAGACGAGTAATCT**CATATGAATATCCTCCTTAG**	EM3333
F671	AAGATGACGACGATGTACAAAAAGTTTATCATAACGTCGCAAATCTCTAAT**GTGTAGGCTGGAGCTGCTTC**	EM3335
R672	CTCGATCCGAGCCGCAAAAGTGTGAAAAAAAGCCAGTTGTGTCGGAATAAA**CATATGAATATCCTCCTTAG**	EM3335

F674	GCGATTAATACTGCGCGTAATATAATTAAAAATCCAGCATTCTCAATCCAT**GTGTAGGCTGGAGCTGCTTC**	EM3337
R675	CCAAAAAGCTGAAAGCTATGGGCGAAATGAAAAACGGCGAAGCGAAGTAAT**CATATGAATATCCTCCTTAG**	EM3337
F707	GAGAAATGATGCAGTGGTGGGCGGACTGGCTTGATGAAAAGGTAGGGTGAT**GTGTAGGCTGGAGCTGCTTC**	EM3339
R708	GGGCGATGAGCGAATTCATTTTAGCGTTTTCCATAGTACGTCTCTTTGCGC**CATATGAATATCCTCCTTAG**	EM3339
F710	ATGGCGGTTACCGACGGTCGAATCAAATCTAAAGCTGGCATGATGCCTCCT**GTGTAGGCTGGAGCTGCTTC**	EM3341
R711	CGGGCGGGCGATCCGTCACTGTTCTTCTGATGACGCGGAGATAACACATCA**CATATGAATATCCTCCTTAG**	EM3341
F713	AAAGCGTGGGCAAAGCAGAAGAGAATAAATCAGATAGCTGGTTTAACTAAA**GTGTAGGCTGGAGCTGCTTC**	EM3343
R714	CACGGTGACGATGGCTTTGCGTACCCGATGTAACGTATTCAGGTGTTCGTA**CATATGAATATCCTCCTTAG**	EM3343
F645	CGCGAATGGCAGGTGGATAAAGCGCGTATCATGAAAAACGCCCATCGTTAA**GTGTAGGCTGGAGCTGCTTC**	EM3345
R646	CCTAATAGAAGTATGAGTGGAAGATGTGTTTATCAGGGATGAGATGGCTTT**CATATGAATATCCTCCTTAG**	EM3345
F677	CTCCTTCTGGCAATGCCGCTTTCCGCCGCCTGGACCGACTGGTTTGCCTGA**GTGTAGGCTGGAGCTGCTTC**	EM3347
R678	GTGAATGTGCAGCGGCTGGAGTTTGGGCGGGGTAAAGTATCAGGAGATTAA**CATATGAATATCCTCCTTAG**	EM3347
F716	CAGTAAACGGCATATTATCAGTGATAGTTCAAACAGTTAGTAGGTAGAAAA**GTGTAGGCTGGAGCTGCTTC**	EM3349
R717	TTTCTGACAGGTTTGTAGAAGCAATTTTGCAGTTTTGGCGCGAAAGGTGAG**CATATGAATATCCTCCTTAG**	EM3349
Primers used for bacterial deletions: Letters in upper case for antibiotics cassette homology		
F398	gc *tctaga* gacgcaagtaacggttgg	pCN2691
R399	gc *gaattc* cgcttgtcacctcttcatgg	pCN2691
F435	atgtcatggtgatgtttgttaagaaagtaaagattgattcattttgaagg GTGTAGGCTGGAGCTGCTTC	CN2785
R436	accaactccatccatgcaacaaatccactacactggataaaattactaaa CATATGAATATCCTCCTTA	CN2785
F748	Ctgaatatgttcaatctgaaactggctgagtatctggtactgtacaacag GTGTAGGCTGGAGCTGCTTC	SC3518

R749	Gcactgtagtggcacagaacttctgcgacctgaccatcctaaatagctgc CATATGAATATCCTCCTTAG	SC3518
F880	aagacctgtaagctcagtggcatggatactattgagctcagaatggcgag GTGTAGGCTGGAGCTGCTTC	SC3722
F882	ccgtctaaatgacggggcgatattaacatgattagaacaagaggaatttt GTGTAGGCTGGAGCTGCTTC	SC3681
F884	aagtcttccggttgtggttgaaaaatatcgttctttaggtatagattcag GTGTAGGCTGGAGCTGCTTC	SC3680
F861	tatattatttccactgtcaggcggaaaatttatagaaaggatacaaacat GTGTAGGCTGGAGCTGCTTC	SC3720
F888	actgagcgatgccgatcatagcaccaattccccgcgacgaatgacgcctg GTGTAGGCTGGAGCTGCTTC	SC3678
R889	cacattgaacggcactgatgattgcgcgttatcgtatcgtgaagcgcctg CATATGAATATCCTCCTTAG	SC3678, 3680, 3681, 3720, 3722
F946	ccgtctaaatgacggggcgatattaacatgattagaacaagaggaatttt CAAGAGGGTCATTATATTTCG	SC3909, 4102
R947	atctgaatctatacctaaagaacgatatttttcaaccacaaccggaagacact CGACATCTTGGTTACCG	SC4102
F948	aagtcttccggttgtggttgaaaaatatcgttctttaggtatagattcag CAAGAGGGTCATTATATTTCG	SC3908
R949	taatgaaatagaattaattttagccccctacacaagtggctgagcttcta CTCGACATCTTGGTTACCG	SC3908, 3909

Primers used for cloning: italics for restriction site upper case for stop codon, underline for 6His		
R874	cg *ctgcag* TTA <u>gtgatggtgatggtgatg</u> ccatgaactgctggtatac	To clone *nleB*$_{IE6}$-6His with *Pst*I site
F875	cg *gaattc* atg attaatcctg ttactaatactc	To clone *nleE*$_{IE6}$ into pSA10 with *EcoR*I site
R876	cg *ctgcag* TTA <u>gtgatggtgatggtgatg</u> ctcaattttagaaagtttattatttatg	To clone *nleE*$_{IE6}$-6His into pSA10 with *Pst*I site
F941	gc *gaattc* tacctaagcgagttcagcgg	To clone *nleB*$_{IE6}$ with its promoter region into pSA10
F528	taa *ggatcc* cgccaccatgg	To clone *mCherry* from pRSET-B into pEGFP-N1 with *BamH*I site
R529	gagtc *gcggccgc* tttacttgtacagctcgtccatg	To clone *mCherry* from pRSET-B into pEGFP-N1 with *Not*I
F982	ga *gctgta* caagtactgcggccgcgactctagatc	To create *Sca*I site instead of stop codon in pMS30
R983	gatctag agtcgcggccgc agtact tgtacagctc	To create *Sca*I site instead of stop codon in pMS30
F807	ggttg *ggtacc* atgattaatcctgttactaatactc	To create both *nleE* $_{IE2}$-*blaM* and *nleE* $_{IE6}$-*blaM* fusions in pHG3760 and pHG3761 respectively, with *KpnI.*
R808	gc *gaattc* cc ctctattttagcaaatttattatttatg	Used to create the *nleE* $_{IE2}$-*blaM* fusion in pHG3760 with *EcoRI*
R811	gc *gaattc* cc ctcaattttagaaagtttattatttatg	Used to create the *nleE* $_{IE6}$-*blaM* fusion in pHG3761 with *EcoRI*
F984	aaa *agtact* cg atg attaatcctg ttactaatactc	To clone *nleE* $_{IE6}$ or *nleE* $_{IE2}$ downstream to *mCherry* with *Sca*I
R985	ataagaat *gcggccgc* TTA *gtgatggtgatggtgatg* ctcaattttagaaagtttattatttatg	To clone *nleE* $_{IE6}$ or *nleE* $_{IE2}$ downstream to *mCherry* with His6 with *Not*I
