# Supplementary figures and images for: The Type III Secretion Effector NleE Inhibits NF-κB Activation
Source: PLoS Pathog. 2010 Jan 29;6(1):e1000743. doi: 10.1371/journal.ppat.1000743 (PMC2813277; doi:10.1371/journal.ppat.1000743)

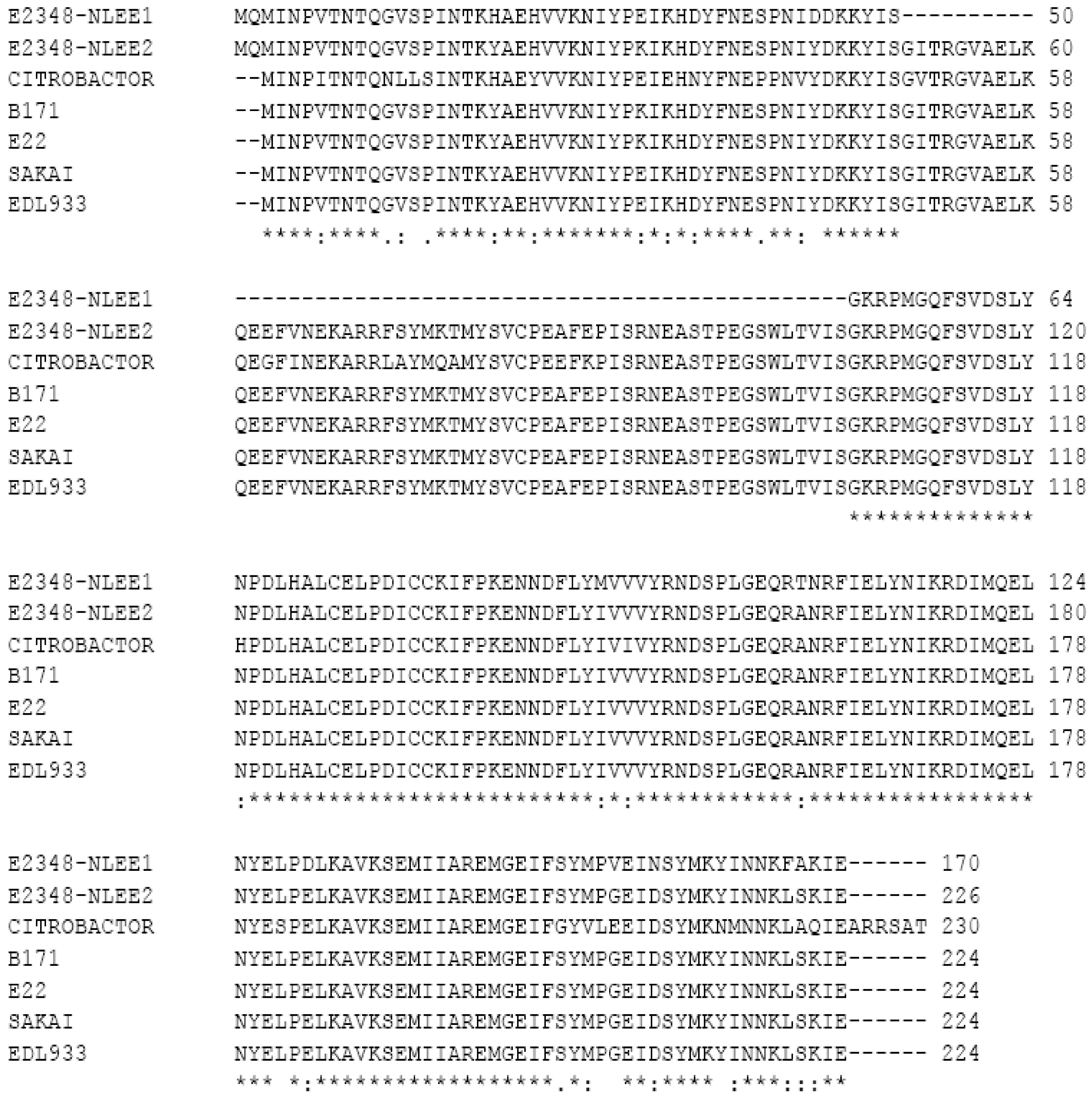

Supplement: Figure S1 — Comparison between different NleE genes of different EPEC and EHEC strains. The E2348 NleEIE6 is indicated as E2348-NLEE2 and NleEIE2 as E2348-NLEE1). Other NleE proteins are those of two EPEC strains (O111 B171 and O103 E22), two EHEC O157 strains (Sakai and EDL933) and Citrobacter rodentium. (1.58 MB TIF) [file ppat.1000743.s004.tif]

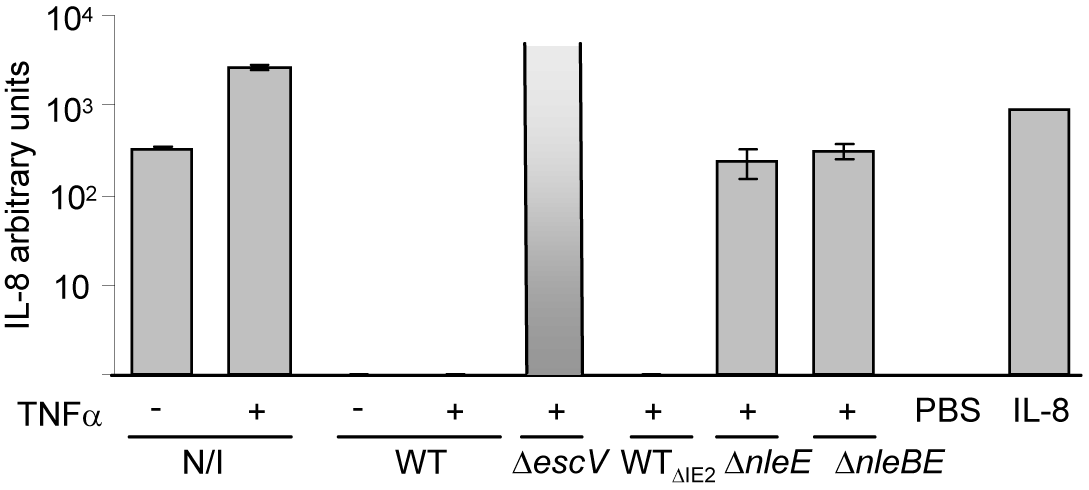

Supplement: Figure S2 — NleE is involved in blocking IL8 secretion. HeLa cells (8×10^4^ per well, seeded in 24-well plate) were infected for 3h with different EPEC strains as indicated or remained uninfected (N/I). After 3.5 h, supernatants were replaced with 300 ul DMEM, 2% FCS, and 50 µg/ml gentamycin with or without 10 ng/ml TNFα. After 16 h, 100 µl of cleared supernatant taken from each well was used for IL-8 measurements using Human CXCL8/IL-8 Quantikine immunoassay assay (R & D), according to the manufacturer's instructions. PBS and IL-8 were used as negative and positive controls for the detection assay. The relative amounts of IL-8 are shown. The experiment was done twice in duplicates and typical results are shown. Standard errors are indicated by bars. In the case of ΔescV (indicated by a vanishing colored bar), the signal was above the upper limit of the detection levels. Untreated and uninfected cells secreted ∼300 units of IL-8, whereas TNFα treatment induced a 10-fold increase in IL-8 secretion (∼3000 units) (Fig. 4B). In contrast, pre-infection with wild-type EPEC or with the ΔIE2 mutant (WTΔIE2), reduced IL-8 secretion below the detection levels, even upon TNFα treatment. Furthermore, the TTSS escV mutant was completely deficient in blocking IL-8 secretion. In conclusion, EPEC strongly reduces IL-8 secretion by a TTSS-dependent mechanism. Importantly, we found that the ΔnleE or ΔnleEB mutants were strongly deficient in blocking IL-8 secretion but not as deficient as the TTSS mutant (escV). These results show that (i) NleE is required for full inhibition of IL-8 secretion and (ii) other putative TTSS effector(s) might function in parallel to NleE to inhibit IL-8 expression and/or secretion. (1.62 MB TIF) [file ppat.1000743.s005.tif]

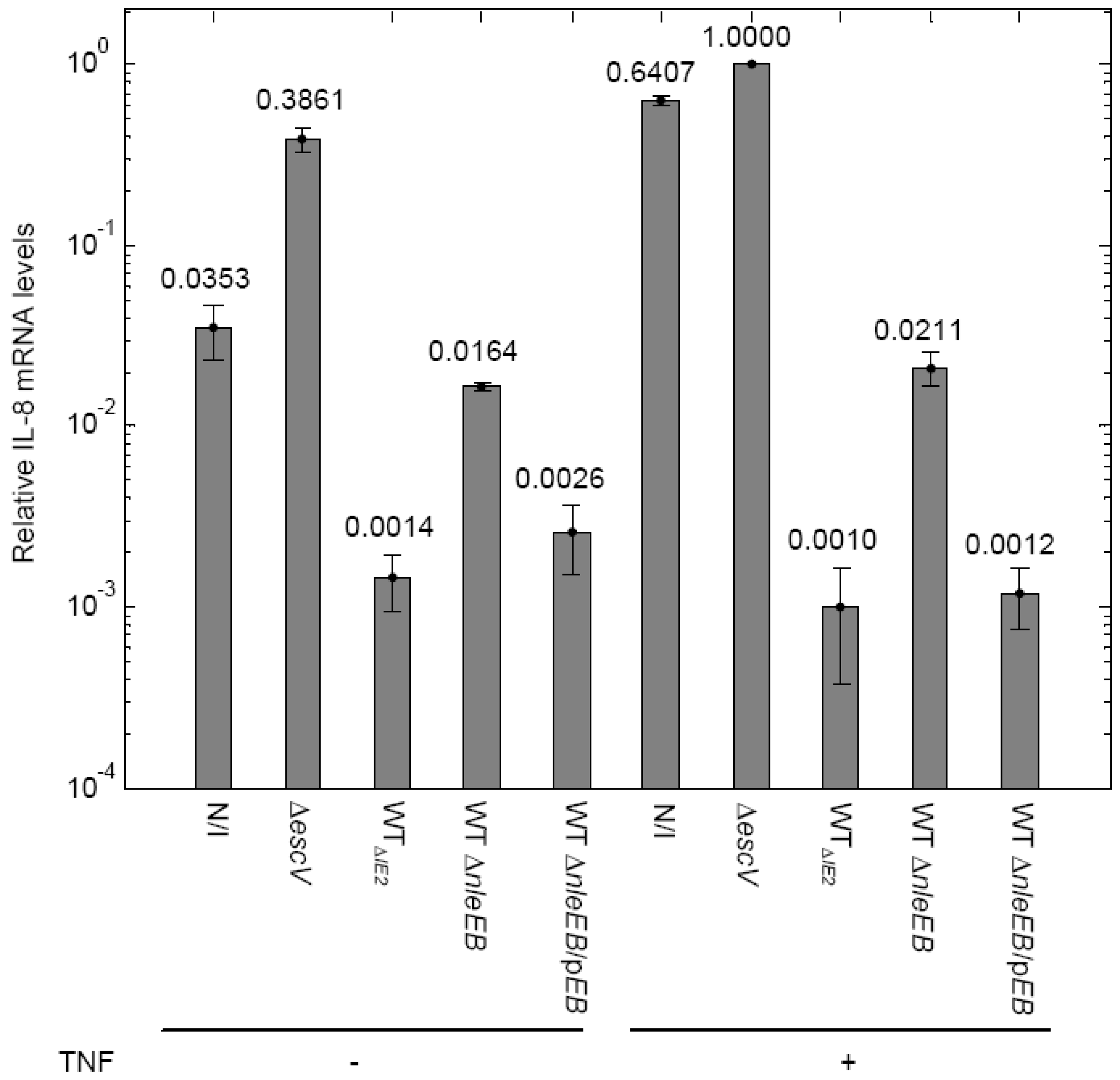

Supplement: Figure S3 — A mutant baring a deletion of nleBE is deficient in repressing self-activated, or TNFα-induced, IL8 repression. HeLa cells were infected with the indicated strains and analyzed as described in Fig. 4B and 4C. To terminate the infection and induce IL8 expression, the medium was replaced with fresh DMEM supplemented with 2% FCS, 100ug/ul gentamicin and with or without 10 ng/ml TNFα and incubated for additional 3 h. Cells were than washed with 2 ml of cold TBS (20 mM Tris-HCl, pH 7.4, 150 mM NaCl), scraped with 1 ml of cold TBS, collected and centrifuged, (800 g, 2 min, 4°C). RNA was extracted using the MasterPure Complete DNA and RNA Purification Kit (EPICENTRE Biotechnologies) and used to synthesize cDNA with the Verso cDNA kit (Thermo Scientific). hHPRT transcript levels were used to normalize total RNA levels in samples. Real time analysis was than conducted using Absolute Blue QPCR SYBR Green (Thermo Scientific) in a real-time cycler (Rotor-Gene 6000, Corbett). The amount of IL-8 mRNA in each strain is shown as a percentage of the level relatively to the transcript levels in the ΔescV mutant. The experiment was done twice in duplicates and typical results are shown. Bars indicate standard errors. (0.44 MB TIF) [file ppat.1000743.s006.tif]

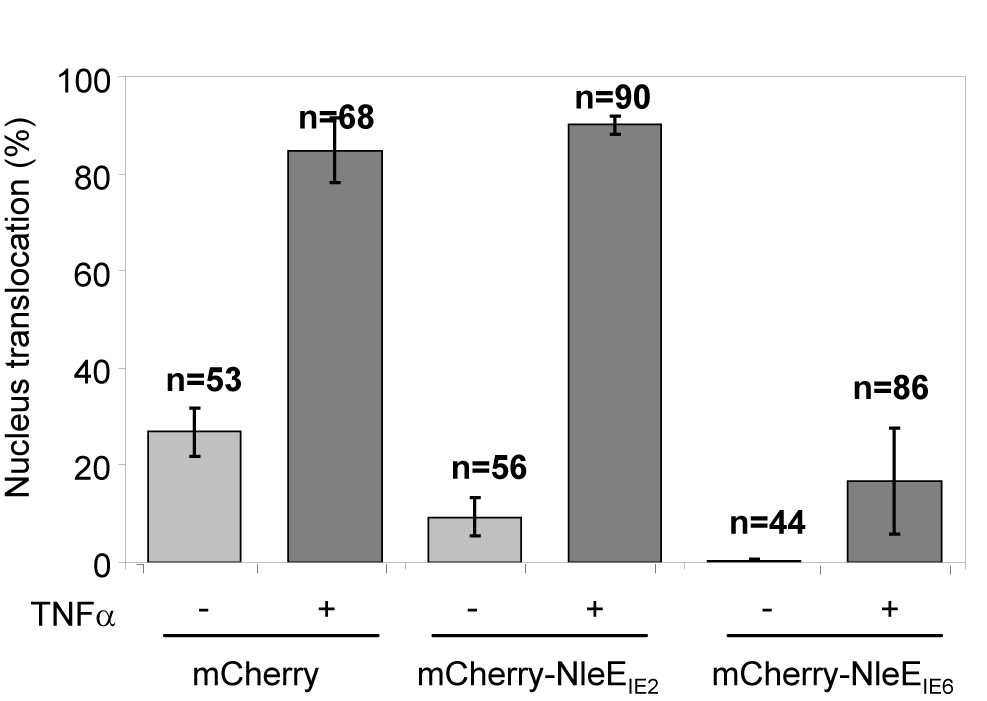

Supplement: Figure S4 — NleEIE2 is deficient in blocking TNF-induced translocation of p65 to the nucleus. HeLa cells were transected with plasmids expressing mCherry, mCherry-NleEIE2 or mCherry-NleEIE6. The expressing cells were treated with TNFα for 1 h, or remained untreated, after which they were fixed and stained with anti-p65. The slides were analyzed by fluorescent microscopy and the percentage of red cells (expressing mCherry or mCherry fused to NleEIE6 or NleEIE2) containing nuclear p65 was determined. The number of cells quantified is indicated and standard errors are indicated by bars. The results show that while NleEIE6 inhibited p65 translocation, NleEIE2 failed to do so. (2.09 MB TIF) [file ppat.1000743.s007.tif]
